# An Environment-Aware Adaptive Data-Gathering Method for Packet-Level Index Modulation in LPWA

**DOI:** 10.3390/s24082514

**Published:** 2024-04-14

**Authors:** Osamu Takyu, Keita Takeda, Ryuji Miyamoto, Koichi Adachi, Mai Ohta, Takeo Fujii

**Affiliations:** 1 Department of Electrical & Computer Engineering, Shinshu University, Nagano 380-8553, Japan; takyu@shinshu-u.ac.jp (O.T.); 22w2062e@shinshu-u.ac.jp (K.T.); 22w2105b@shinshu-u.ac.jp (R.M.); 2 Advanced Wireless & Communication Research Center, University of Electrocommunications, Tokyo 182-8585, Japan; adachi@awcc.uec.ac.jp; 3 Department of Electronics Engineering and Computer Science, Fukuoka University, Fukuoka 814-0180, Japan; maiohta@fukuoka-u.ac.jp

**Keywords:** LPWA, PLIM, mathematical optimization

## Abstract

Low-power wide-area (LPWA) is a communication technology for the IoT that allows low power consumption and long-range communication. Additionally, packet-level index modulation (PLIM) can transmit additional information using multiple frequency channels and time slots. However, in a competitive radio access environment, where multiple sensors autonomously determine packet transmission, packet collisions occur when transmitting the same information. The packet collisions cause a reduction in the throughput. A method has been proposed to design a mapping table that shows the correspondence between indexes and information using a packet collision minimization criterion. However, the effectiveness of this method depends on how the probability of the occurrence of the information to be transmitted is modeled. We propose an environment-aware adaptive data-gathering method that identifies the location of factors affecting sensor information and constructs a model for the probability of the occurrence of sensor information. The packet collision rate of the environment-aware adaptive data-gathering method was clarified through computer simulations and actual experiments on a 429 MHz LPWA. We confirm that the proposed scheme improves the packet collision rate by 15% in the computer simulation and 30% in the experimental evaluation, respectively.

## 1. Introduction

In recent years, low-power wide-area (LPWA) communication technology has attracted attention as a wireless sensor network for the internet of things (IoT) due to its long-range capability and low power consumption. LPWA operates without a license, enabling the deployment of wireless networks virtually anywhere [1]. In particular, the event detection scheme that detects the environmental changes in IoT has been considered in [2]. In the IoT for agriculture, the event detection based on machine learning is considered in [3]. For the IoT of the inside of a car, the error detection of the equipment system is considered in [4]. For monitoring the water level, the highly accurate error detection based on a machine learning scheme is proposed in [5]. Event detection from the open sky using an unmanned aerial vehicle is proposed in [6]. Various frame works for highly accurate event detection are proposed in [7]. In addition, the process flow of event detection with the analysis of the gathered data is shown in [8]. The analysis of time and frequency domains for the robustness of noise is proposed and thus the detection accuracy is improved in [9]. Event detection based on the compressed sensing is proposed in [10]. The variable sampling rate adapted to the conditions of events is considered in [11]. However, as the event or the change in the monitored environment occurs, a lot of sensors recognize it and then access the channel, simultaneously. The packet collisions frequently occur and thus it is quite difficult to gather the sensing data [12]. In addition, LPWAs have the limitation of a data transmission duty cycle to secure a time for other systems to gain access. However, there is also the problem that the limitation of duty cycle reduces throughput.

Various schemes for reducing the packet collision and improving the packet delivery rate have been considered. The performance of LoRa under the industrial application with various algorithms of the adaptive data rate (ADR) is clarified in [13]. When the parameters of the LoRa format, such as packet length and spreading factor, are changed, the packet error rate is evaluated [14]. In the dense sensor nodes, the suitable spreading factor assigned to each sensor in accordance with the criterion of maximal delivery rate is considered [15]. In addition, in the dense sensor nodes, the adaptive switching of spreading factor and transmit power control is proposed for improving the packet delivery rate in [16]. The avoidance of packet collision based on the prediction of packet access is considered in [17]. The construction of a transmitting packet using the period of packet transmission is considered for avoiding the packet collision in [18]. For increasing the throughput, the interleaved chirp pattern of LoRa modulation is considered in [19]. In the transmission range under the 1 km, the packet collisions frequently occur and then the packet retransmission in the application layer is considered in [20]. The distributed control of the packet access parameters improves the packet delivery when late in [21].

One method to enhance the throughput of LPWA within the confines of the duty cycle limitation is through packet-level index modulation (PLIM) [22]. PLIM maintains the packet format while dynamically adjusting the channel and timing of packet transmission according to the information to be transmitted. This effectively utilizes the idle periods created by duty cycle limitations as indices for information transmission, thereby expanding throughput. Additionally, as PLIM adheres to the LPWA wireless standard, it can seamlessly integrate with existing LPWA deployments.

The conventional schemes for avoiding packet collision and compensating the degradation of the delivery rate assume the periodic packet access or the ability to directly controlling the parameters of the packet access. However, in the PLIM, the packet access depends on the sensing information. The conventional scheme cannot directly apply to the LoRa with PLIM. Therefore, the scheme for suppressing the packet collision as well as applying the LoRa PLIM is required.

We consider the application of PLIM in a competitive wireless environment where multiple sensors can transmit packets simultaneously on the same channel and with the same timing. When multiple sensors use PLIM to transmit identical information, they concurrently send packets over the same transmission channel and timing, resulting in packet collisions. Therefore, the mapping table, showing the correspondence between transmitted information and indices, is adapted for each sensor, thereby mitigating packet collisions. A method has been proposed to design a mapping that minimizes the packet collision probability by mathematical optimization [23]. Establishing a model of packet collision probability necessitates understanding the statistical trends of the sensor information to be transmitted beforehand. To achieve this, a method has been proposed that uses previously collected data and prior observations to construct a model of the probability of information occurrence [23]. However, if the information pattern transmitted by each sensor follows a uniform probability distribution, there is a chance that other sensors may select the same index, thereby limiting the effectiveness of packet collision suppression.

In this study, our focus is on identifying the factors influencing each piece of sensor information to understand the statistical trends of prior sensor data. While our explanation primarily pertains to radio sensors, the methodology is applicable to other phenomena that exhibit spatial spillover. Radio sensors are being explored for frequency-sharing applications [24]. In the context of radio sensors, when a radio source emits a signal, the sensor measures the receive signal strength indicator (RSSI) at its location. Subsequently, each radio sensor transmits the RSSI to a gateway (GW) using PLIM. The RSSI observed by each radio sensor varies depending on the location of the radio source. Therefore, we analyzed the statistical trend of RSSI by limiting the presence of radio sources to a narrower area within the overall observation range. Consequently, RSSI tends to exhibit a narrow spread and concentrate around a specific value. Therefore, we analyzed RSSI trends using radio sensors. Here, we divided the entire observation space into uniformly sized sections to form areas. Therefore, the statistical trend of RSSI for each sensor was analyzed according to the presence of radio sources in each area. Upon identifying the area where the radio source was located, the corresponding RSSI trend was used to design the PLIM mapping table. Therefore, each radio sensor focuses on a specific RSSI value and rarely sends other RSSI values to the GW. The vacant indices, which are not used for PLIM, are secured and then used by other sensors, thereby suppressing packet collisions. In the proposed method, the position estimation of the radio source and the selection of a suitable mapping table corresponding to the estimated position are iteratively used. Therefore, the proposed method adaptively switches the mapping table based the radio environment, referred to as an environment-aware adaptive data-gathering method. In this research, the packet collision rate is evaluated by computer simulation to elucidate the effect of reducing the packet collisions using the environment-aware adaptive data-gathering method. Furthermore, the environment-aware adaptive data-gathering method was implemented using 429 MHz-LoRa/FSK modules, and its effects were also assessed in the experimental evaluation.

The difference among our papers [22,23] and this paper is shown as follows. In Ref. [22], we initially proposed the packet-level index modulation. We did not consider the packet collision under the competitive wireless access environment among multiple sensors. Ref. [23] proposed the construction of the mapping table between the indexes, and the sensing information was constructed to minimize the packet collision rate in the PLIM. It confirmed the effect of reducing packet collisions in the computer simulation. However, this has not been confirmed in an experimental evaluation with the actual wireless equipment. In addition, the effect of reducing the packet collisions by the proposed construction of mapping table depends on the tendency of sensing information. Ref. [23] did not consider this in detail. This paper pays attention to the relationship between the position of the event source and the statistical tendency of the sensing information. It proposes the adaptive data-gathering scheme based on the positioning of the event source. In addition, this paper clarifies the effect of the optimal mapping table in an experimental evaluation with the actual wireless equipment, which is 429 MHz LoRa/FSK.

The main contributions of our paper are shown below.

- We propose the adaptive data-gathering scheme in which the mapping table of PLIM is adaptively changed in accordance with the position of event source. The proposed adaptive data gathering can effectively reduce the packet collisions.
- We implement the proposed adaptive data-gathering scheme in the actual equipment of 429 MHz LoRa/FSK. In the practical experiment, the effects of optimizing the mapping table of PLIM and the adaptive data-gathering scheme are clarified.
- In the computer simulation and the experimental evaluations, we confirm the improving effect of the proposed adaptive data-gathering scheme in terms of the reduction in packet collision rate and the packet delivery rate.

The necessity of the experimental evaluation is shown as follows. In computer simulation, the ray-tracing simulator is used for simulating the radio environment. As the number of reflections becomes large, its computational complexity is explosively increased and thus the results cannot be obtained within a practical and finite time duration. Instead, the simple simulation with a smaller number of reflections is used. However, a mismatch between the simple simulation and the actual radio environment occurs. In the proposed data-gathering method, the spatial correlation of the radio environment is used and thus the highly accurate simulation of the radio environment is necessary. Therefore, the experimental evaluation with the actual radio sensor measuring the RSSI can clarify the practicality of the proposed method. In the evaluation of Ref. [23], the packet access from each sensor to the GW is assumed as the ideal model with no packet loss. The LPWA is the wide area network and thus the sensor located in each site suffers from the various obstacles around it. Therefore, the channel model is different for each sensor. For simulating the highly accurate wireless communication, the channel model is constructed sensor by sensor and thus the process time of evaluation and constructing the program are significantly large. In the experimental evaluation with the packet transmission based on LPWA, the particular channel model in each sensor is considered and then the effect of the proposed data-gathering method can be evaluated. In addition, the processes of the proposed data-gathering method are composed of the positioning of the radio source, the selection of the suitable mapping in the PLIM, the informing from the GW to each sensor about the selected mapping, and the switch of the mapping in each sensor. These consume a certain amount of time as the processing time. As the adaptation of the proposed data-gathering method to the radio environment is evaluated, the processing time should be considered and thus the actual implementation of the proposed method is necessary.

## 2. System Model

In the sensor networks considered in this study, there was one GW and multiple sensor nodes. Each sensor converted its observation information into digital data and notified the GW. The GW periodically broadcasted time synchronization signals to all the sensors, ensuring time synchronization across the network. Each sensor transmitted its observation information once at periodic intervals, defining the transmission cycle as a frame. The observation information was encapsulated within packets containing necessary control information for communication. Therefore, each sensor sent a packet only once per frame. However, the packet access timing could be chosen arbitrarily as long as it fell within one frame time; the frame time was assumed to be significantly longer than the packet length.

LoRa is assumed to be the transmission method for LPWA, where a chirp pattern corresponding to the digital information is selected for transmission. This modulation is referred to as LoRa modulation. The duration of the chirp pattern is determined by the spreading factor (SF). As the SF increases, the symbol length of the chirp pattern expands, and the number of chirp pattern types increases. Consequently, the number of bits that can be transmitted per symbol also increases. Furthermore, as the energy per symbol is enlarged, the transmission distance is increased. However, when the SF is set to 1, two different frequencies are switched for and sensors autonomously select one channel each, which is equivalent to Frequency Shift Keying (FSK).

### 2.1. Packet Level Index Modulation (PLIM) [22]

**Figure 1:**
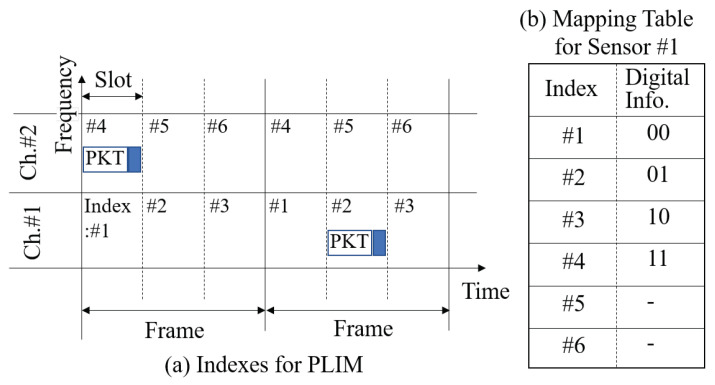
Image of PLIM.

PLIM is an index modulation technique where the index is determined by the transmission timing and frequency channel of the packet. Figure 1a shows the relationship between the channel and time. The frame is divided into short, equally spaced segments called slots, with each slot being shorter than the frame length but longer than the packet length. The frame start time for each sensor is detected using a synchronization signal from the GW. Each sensor sets the waiting time for packet transmission in slots from the frame’s start time, allowing them to select a specific slot for packet transmission by adjusting their transmission waiting time. Moreover, multiple orthogonal frequency channels are prepared, and sensors autonomously select one channel each. Consequently, the total number of indices that can be formed is the product of the total number of frequency channels and the total number of time slots. The digital information is assigned to the index formed in this manner, a process called mapping table. Figure 1b presents an example of a mapping table. Each sensor can independently set its own mapping.

Information transmission in PLIM operates as follows: The sensor constructs a digital pattern, which is a combination of multiple pieces of digital information to be transmitted. In the case of transmitting two bits, there are four possible patterns: 00, 01, 10, and 11. Subsequently, the sensor selects the index corresponding to the digital pattern by referring to the mapping table. The packet is then transmitted in the timeslot and frequency channel indicated by the selected index. Upon reception, the receiver identifies the number of received frequency channels and the timing of reception. Since time synchronization between the receiver, GW, and each sensor node has been established, the difference between the start time of the frame and the arrival time of the packet indicates the timeslot selected by the transmitter, i.e., the sensor. The index is obtained from the detected frequency channel and timeslot. The GW refers to the mapping table used by the sensor whose ID is recognized from the header of the received packet. It then identified the digital pattern corresponding to the received index, thus receiving this digital pattern as the digital information from the sensor.

In PLIM, besides the packet payload, additional information can be transmitted using the index, thereby expanding the throughput. Furthermore, the modulation format of the packet does not need to be altered, making it compliant with the LoRa standards.

### 2.2. Optimization of Mapping in PLIM [23]

One of the challenges of PLIM is the occurrence of packet collisions when multiple sensors select the same index, resulting in missing information. To mitigate packet collisions, the construction of the mapping table based on mathematical optimization techniques has been constructed. A quadratic programming problem is formulated by deriving the probability of packet collision between any two sensors and using the mapping pattern rules in PLIM as constraints. The specific model equation is as follows:

(1) $$\min\;\sum_{k=1}^{K}\sum_{i_{1}=1}^{I}\sum_{i_{2}=i_{1}+1}^{I}\sum_{j_{1}=1}^{J}\sum_{j_{2}=1}^{J}P_{i_{1},j_{1}}P_{i_{2},j_{2}}x_{i_{1},j_{1},k}x_{i_{2},j_{2},k}$$

$$\begin{array}{c} \mathrm{subject}\;\mathrm{to}\;\;\;\;x_{i,j,k}\in \left\{0,1\right\}\;\;\;\;\;\forall i\in I,\forall j\in J,\forall k\in K \end{array}$$

(2) $$\begin{array}{c} \sum_{k=1}^{K}x_{i,j,k}=1\;\;\;\;\;\forall i\in I,\forall j\in J \end{array}$$

(3) $$\begin{array}{c} \sum_{j=1}^{J}x_{i,j,k}\leq 1\;\;\;\;\;\forall i\in I,\forall k\in K \end{array}$$

(4) $$\begin{array}{c} 1\leq \sum_{j=1}^{J}\sum_{i=1}^{I}x_{i,j,k}\leq I\;\;\;\;\;\forall k\in K \end{array}$$

Here, *i* indicates the sensor number, *j* indicates the digital pattern number assigned by a particular decimal number, and *k* indicates the index number, respectively. Additionally, *I*, *J*, and *K* denote the total number of sensors, the total number of digital patterns, and indices, respectively. $P_{i,j}$ indicates the probability that the *i*th sensor will send the *j*th digital pattern. When $x_{i,j,k}$ is one, the *k*th index is assigned to the *j*th digital pattern of the *i*th sensor. Conversely, zero indicates no assignment. Note that $x_{i,j,k}$ is the optimization variable.

Equation (2) indicates that each digital pattern is assigned an index for each sensor. Equation (3) indicates that at most, one digital pattern is assigned to one index at each sensor. Equation (4) also ensures that each index is assigned at most one digital pattern for any one sensor, and at most up to the number of sensors. The optimization solver can be used to determine the design variables $x_{i,j,k}$.

In Ref. [23], the construction of mapping in the PLIM is modeled as the quadratic programming problem. The Gurobi Optimization [25], which is the solver of the quadratic problem, is used for deriving the optimal variables [23]. We also use the Gurobi Optimization as the solver of the quadratic programming problem. The computational cost of constructing mapping table is more significant as the number of variables becomes large [23]. Therefore, we also use the quasi-optimization solution with low complexity, as Ref. [23] also uses it.

The model equation requires a prior estimation of $P_{i,j}$. Estimation methods include prior observations and analogous methods based on previously gathered results. Here, we consider a case where a particular digital pattern in $P_{i,j}$ exhibits a higher probability of occurrence than the other patterns. Maintaining a low collision probability entails assigning a digital pattern with low occurrence probability to the index assigned to the digital pattern with high occurrence probability. This concept yields the mapping table derived from optimal design, which can effectively suppress packet collisions. Conversely, if the occurrence probability of any digital pattern is uniform, the probability of selecting any index is not low, even with the mapping changes. Therefore, the probability of multiple sensors selecting the same index is high, limiting the effectiveness of collision suppression. Therefore, when the occurrence probability of a particular digital pattern is high, the collision-suppression effect of the mapping design can be enhanced. In this study, we established an environment-aware adaptive data-gathering method where the occurrence probability of a digital pattern is concentrated on a specific pattern.

### 2.3. An Environment-Aware Adaptive Data-Gathering Method

This study assumes that a sensor observes the RSSI of a radio wave and transmits it via PLIM. The same concept can be applied to cases where temperature, humidity, etc., are transmitted. The magnitude of RSSI observed by the radio sensor is quantized at regular intervals, making RSSI a discrete value. Each discrete value corresponds to a digital pattern of PLIM. Therefore, a unique index is assigned to each discrete value, and then the RSSI is transmitted by PLIM. Each discrete value of RSSI is associated with a decimal number, referred to as the RSSI number.

By uniformly placing sensors in the observation area and measuring RSSI, the spatial spread of radio waves emitted by a radio source is observed. Radio wave propagation is subject to shielding effects from buildings and attenuation corresponding to the propagation distance [26]. Therefore, the shielding effect of each building differs depending on the position from which the radio waves are emitted, resulting in inherent variability in radio propagation depending on the location of the radio source. The RSSI dataset, which aggregates the occurrence probability of the RSSI observed by each sensor, can be considered to have specificity depending on the location of the radio source. A method using this RSSI-spatial specification for location positioning has also been investigated [27]. In this study, we develop a probabilistic model of RSSI corresponding to the location of the radio source.

**Figure 2:**
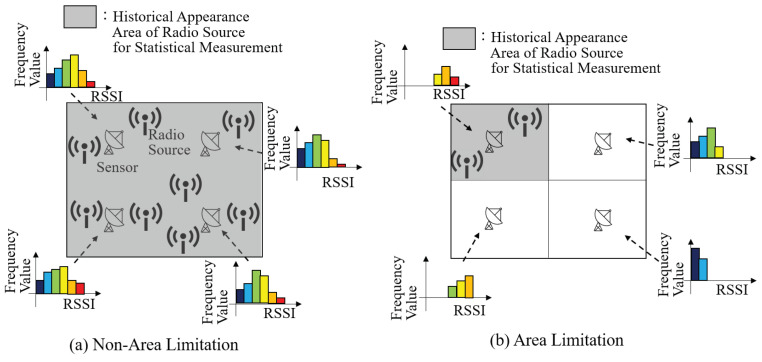
Stochastic tendency of detected RSSI depending on concentration level of appearing radio source. The colors indicate the level of RSSI.

Figure 2 shows the past locations of radio sources and the probability distribution of RSSI observed by a certain single sensor. Note that each figure for a radio source shows the cumulative past locations, and these sources do not emit radio waves simultaneously. Figure 2a shows the case where radio sources are uniformly distributed within the observation range for evaluating the frequency distribution to determine the probability of RSSI for each sensor. Conversely, Figure 2b shows the case where radio sources are uniformly distributed within the observable range for evaluating the frequency distribution to determine the probability of RSSI for each sensor. This represents a case where the occurrence range of past radio sources is restricted to an area smaller than the observation range. From the figures, it is evident that the probability distribution of RSSI in Figure 2b converges more narrowly than that in Figure 2a. Therefore, if the range of occurrence of a radio source can be limited to a certain area, the probability distribution of transmitted information can be constrained more narrowly.

Therefore, we propose the following environment-aware adaptive data-gathering method: We divided the observed space of radio waves into equally sized small areas. Then, we recorded the RSSI of each sensor corresponding to the occurrence of radio sources at various locations within each area. The RSSI recorded by each sensor was analyzed using histogram analysis to obtain the probability of occurrence of each RSSI value. Consequently, a probability model for the occurrence of the RSSI for each sensor can be obtained for each area. This provides the prior RSSI occurrence probability necessary for designing the optimal mapping. Next, the optimal mapping was derived based on the reference [23]. As a result of these processes, the optimal mapping was established for each area. The mapping corresponding to each area was recorded for each sensor and GW.

**Figure 3:**
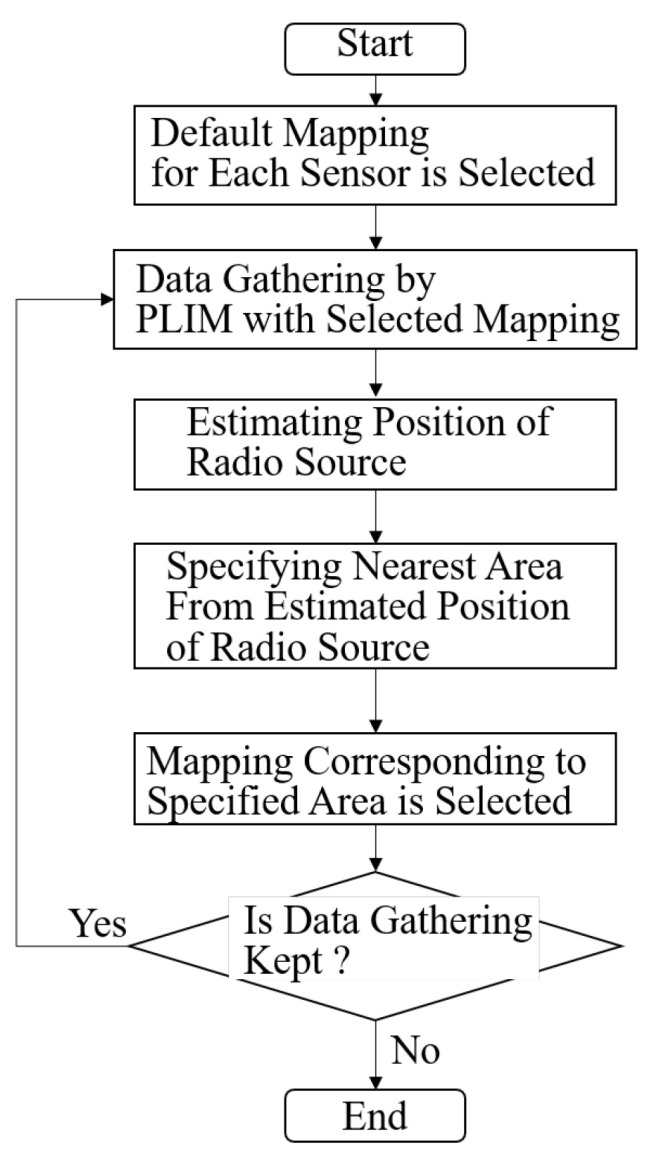
System flow of environment-aware adaptive data-gathering method.

For the information gathered by PLIM, mappings are selected adaptively according to the process flow shown in Figure 3. As we can see this figure, the selecting mapping is composed of the iterative process. Each sensor first informs PLIM of its observed RSSI using the default mapping. Here, a random mapping table is used as the default mapping, constructed by establishing random correspondence between the RSSI numbers and the indices. The RSSI gathered by all sensors is then utilized by the GW to estimate the area where the radio source is located. In the method of estimating the radio source’s location, the sensor observing the maximum RSSI can be considered as the location of the radio source, or the center-of-gravity addition method [28] can be used. The estimated area where the radio source exists is then communicated from the GW to each sensor. Subsequently, the GW and each sensor select a mapping designed according to the RSSI probability distribution for each area. The selected mapping is used to notify PLIM of the RSSI. This series of processes is then repeated: radio source location estimation, area location estimation, GW notifying each sensor of the estimated area, switching the mapping, and PLIM transmission.

By proceeding the iterative process of the proposed method, it becomes possible to select an appropriate mapping table according to the radio source and transmit it by PLIM. Due to the limited range of radio sources, packet collisions among sensors can be suppressed using a mapping table with an optimized design that uses the converged RSSI distribution. Additionally, as the location of the radio source is monitored, mapping can be adjusted to accommodate changes in the radio environment resulting from the movement of the radio source. Therefore, the success rate of sensor information collection can be maintained at a high level.

The proposed method was unable to prevent packet collisions resulting from multiple sensors transmitting packets with the same index when using the initial default mapping. This resulted in missing RSSI data, leading to errors in the estimation of radio source locations. To mitigate these errors, the area can be expanded within a certain range to provide a margin against location estimation errors. However, enlarging the area reduces the collision-avoidance efficacy of the mapping design as the probability distribution of RSSI widens. Hence, there exists a tradeoff between the margin for position estimation error and the collision avoidance effect of the mapping design.

## 3. Implementation of Environment-Aware Adaptive Data-Gathering Method to 429 MHz LoRa/FSK

**Figure 4:**
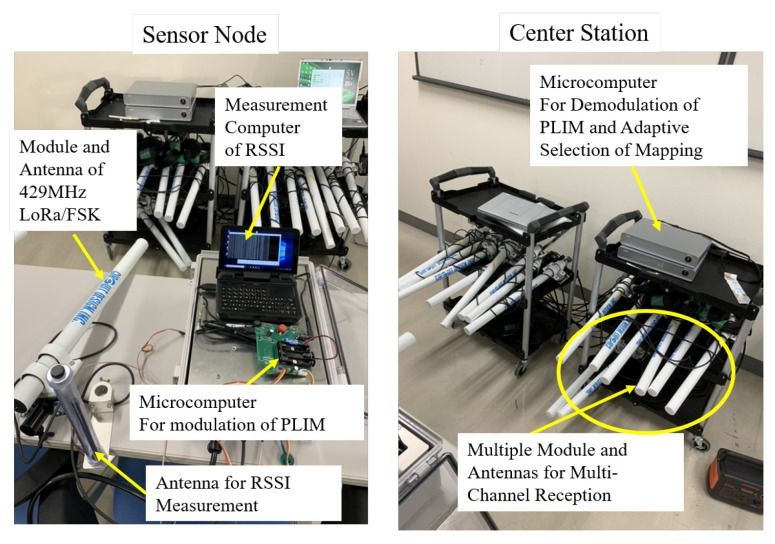
Overview of constructed method to 429 MHz LoRa/FSK.

The proposed environment-aware adaptive data-gathering method was implemented using a 429 MHz LoRa/FSK. Figure 4 shows an overview of the proposed method for 429 MHz LoRa/FSK. The 429 MHz LoRa/FSK utilizes a LoRa-BAR module manufactured by Circuit Design, Inc. This module integrates an antenna and a modulation, and demodulation circuity, allowing it to emit radio waves in compliance with the LoRa standard.

The sensor was equipped with a microcontroller for PLIM control. This microcontroller managed the LoRa-BAR channel and transmission timing to realize the PLIM. Additionally, the sensor featured an antenna for RSSI observation and a universal software radio peripheral for RSSI calculation. Based on the RSSI value obtained, the sensor referred to the mapping table to select an appropriate index. Subsequently, it determined the transmission timing and channel for the packet corresponding to the selected index and transmitted the packet accordingly. A mapping table was designed, with one assigned to each area, where the radio source was present. Consequently, each sensor has a mapping table equal to the number of areas. The GW stored a mapping table recorded for each sensor categorized by area. By employing methods like the maximum RSSI method or the center-of-gravity addition [28], the GW determined the mapping for each sensor corresponding to the identified area. Furthermore, it informed all the sensors within the identified area accordingly. Since each sensor’s LoRa-BAR supported half-duplex communication, the GW received and processed data from each sensor at the time of the announcement. Time synchronization was established during periodic beacon transmissions. Subsequently, each sensor selected a mapping corresponding to the area notified by the GW and used it for PLIM modulation.

The GW has a number of LoRa-BARs equal to the number of channels, allowing the simultaneous reception of multiple channels. All LoRa-BARs are connected to a microcontroller for PLIM demodulation. When a LoRa-BAR successfully receives a packet, it notifies the source information and the received data to the PLIM demodulator microcontroller. Since each LoRa-BAR corresponds to one channel, the LoRa-BAR that notifies the information is distinguished, and the channel number used by the sensor to transmit the packet. Next, the time of data arrival of the information to the microcontroller was determined as the packet arrival time, and the time slot in which the sensor transmitted the packet was identified. Identification of the channel number and time slot helps to identify the index that the sensor chooses in its transmission. The sensor that sends the packet is identified by the ID provided in the packet header. The mapping table for the identified sensor is then used to identify the transmitted information corresponding to the index, and PLIM demodulation is completed. In this implementation, transmission and reception by PLIM modulation, estimation of the area of the radio source, notification of the estimated area to all sensors, and switching of the mapping table by each sensor according to the notified area were completed within the frame time length defined by PLIM. When these flows were repeated, the GW tracked the position of the radio source. Therefore, the proposed method can select a suitable mapping table for a radio environment that is affected by the mobility of the radio source.

## 4. Computer Simulation

**Figure 5:**
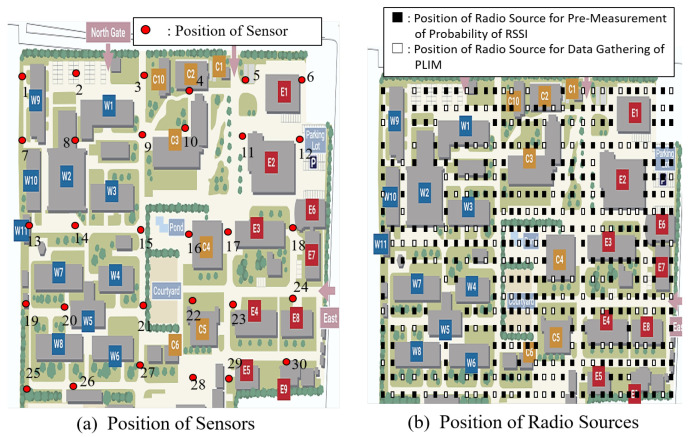
Assumed wireless sensor networks for simulation. In (**a**), the number of sensors is 30, and in (**b**), the numbers of radio sources for pre-measurement of probability of RSSI and for data gathering of PLIM are 240 and 171, respectively.

A simulation is conducted assuming a radio sensor that measures the RSSI of radio waves emitted by 920 a band LPWA source. Figure 5 presents an image of the proposed sensor network. Thirty sensors were deployed on the site. Radio sources were placed uniformly and randomly on the site, and the sensors measured the RSSI of the radio waves emitted by the sources. A three-dimensional map of the site was applied to a ray-tracing simulation, and the RSSI at the point where each sensor is placed was determined by the radio sensors. The ray-tracing simulation assumed four reflections, zero refractions, and one diffraction. The measured RSSI was considered to be the RSSI measured by the sensor. Each antenna was assumed omni-directional. The arrangement of the radio sensors and sources is shown in Figure 5. Among the 30 sensors, the number of terminals transmitting in the same frame in PLIM was set from 4 to 8. When *x*(=4, 5, 6, 7, 8) sensors compete for access in the same frame, the site is divided into *x* equal parts, and one sensor is randomly selected from each divided area. Of the total radio sources (411 points), 240 points were used as radio emission sources to determine the probability of RSSI generation for each sensor in the proposed method. The remaining 171 points were used to evaluate the packet collision rate when gathering RSSI by PLIM with a generated radio source. Note that two or more radio sources do not emit radio simultaneously; instead, a single radio source emits waves each time. The proposed radio source is divided into nine equal parts, and the positioning method used was center-of-gravity addition. There were a total of 10 values and 10 indexes.

**Figure 6:**
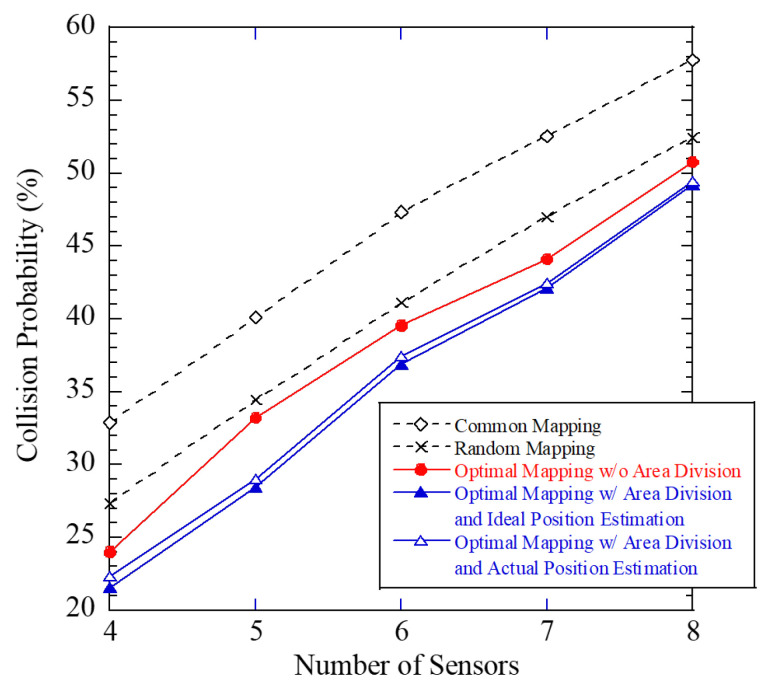
Performance between number of sensors and packet collision rate.

The packet collision probability against the number of sensors is shown in Figure 6. The figure shows four mapping tables: Common mapping, random mapping, optimal mapping without area division, and optimal mapping with area division. In a common mapping table, all sensors use a single common mapping table. In the random mapping table, the correspondence between the information and index is randomly constructed for each sensor. In the optimal mapping table without area division, the construction of the mapping table is based on minimizing the occurrence probability of packet collisions using the pre-measured tendency of RSSI without area division of the radio source. By contrast, the optimal mapping table with area division is based on the area division. In the optimal mapping table with area division, we also show the two results when the area of the radio source is estimated without error and when it is estimated. From the figure, the optimal mapping table with area division and ideal estimation achieves a 15% reduction in the packet collision rate than the common mapping table. Additionally, the optimal mapping table with area division and the actual estimation achieved a 13% reduction. Moreover, the optimal mapping table with area division and actual estimation can achieve about 5% drop from that with area division and ideal estimation. The proposed method effectively suppressed packet collision probability.

## 5. Experimental Evaluation

An experiment was conducted to clarify the accuracy of the information gathered by the 429 MHz LoRa/FSK implementing the proposed method. In this study, we observed RSSI using a radio wave sensor in two wireless environments. The first was to measure the RSSI of radio waves emitted by a 5 GHz wireless LAN access point in a typical indoor environment. The second measured the RSSI of radio waves emitted by a 5 GHz wireless LAN access point in an electro-magnetic anechoic chamber. In the latter case, when the emitted radio wave arrives at the wall surface, it is completely absorbed. Radio wave reflections occur from devices in the same room; therefore, radio wave reflections cannot be completely suppressed. However, the radio waves observed by the sensor were in a radio environment where direct waves were dominant.

**Figure 7:**
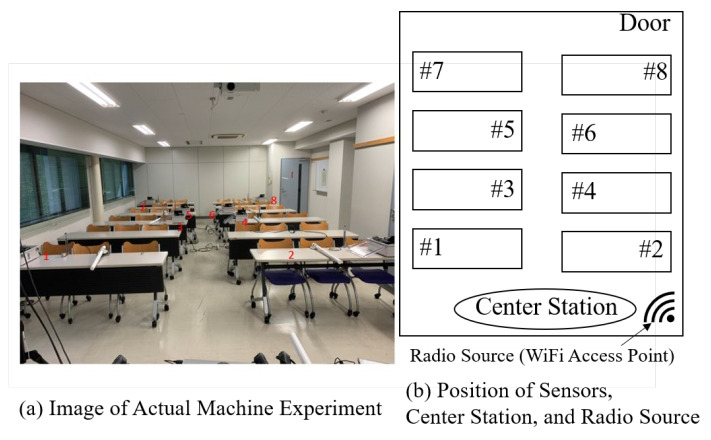
Arrangement of sensors in Experiment 1.

Figure 7 shows the arrangement of the sensors in Experiment 1 and their images. Eight sensor terminals were used in Experiment 1. Each sensor observes the RSSI of the radio waves emitted by the 5 GHz Wi-Fi and notifies the GW via PLIM. The RSSI is quantized at 4 dB intervals from−94 dBm, the minimum value, and quantized into 10 units. In this experiment, both in estimating the prior probability of RSSI occurrence and in aggregating RSSI information using PLIM, the radio sources were assumed to be in the same location. In the trend analysis to determine the probability of RSSI occurrence, the radio source communicated data for 10 consecutive min. Each sensor repeatedly measured the RSSI of radio waves emitted by the source at regular time intervals. The RSSI measured by each sensor was subjected to a histogram analysis to determine the frequency of occurrence of each quantized RSSI, and the probability of occurrence of quantized RSSI was calculated. We repeated this process for each radio source to obtain the occurrence probability of RSSI for each radio source. In other words, we created a database showing the relationship between the occurrence probability of RSSI for each sensor corresponding to a radio wave source. Then, using the occurrence probability of RSSI corresponding to each radio source, we designed a mapping that minimizes the packet collision rate using the proposed method. Through this process, optimal mapping for each sensor was designed based on the location of each radio source. The designed mapping is pre-recorded in the GW and each sensor and used in PLIM.

The parameters used in Experiment 1 are listed in Table 1. A total of 10 indices were used: 1 frequency channel with 10 time slots. To evaluate the packet delivery rate (PDR), the radio sources communicated data continuously for 10 min. During this time, each sensor sent one RSSI measurement and notification to the GW every 5 s. Therefore, the RSSI data were sent to the GW 120 times during the measurement period, and the PDR was evaluated. To confirm the effectiveness of the optimization, we assumed that the location of the radio source was known. Therefore, the proposed method uses an optimal mapping that minimizes the collision probability corresponding to the radio source.

**Figure 8:**
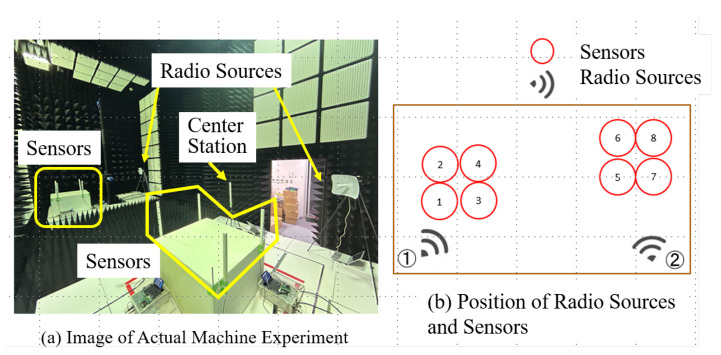
Arrangement of sensors in Experiment 2.

Experiment 2 was conducted in an electromagnetic anechoic chamber with a sensor network and Wi-Fi as the radio source. Figure 8 shows the actual locations of the radio sources and sensors. The radio sources were placed at two locations, each with a radio source device. However, these two radio sources did not emit radio waves simultaneously. In other words, when one radio wave was emitted, the other ceased emitting radio waves. Thus, there was no physical movement of the radio sources and no misalignment between them. Following the same procedure as in Experiment 1, a preliminary trend analysis of radio wave emissions were performed at the placement location of one radio wave source, and measured the RSSI. This process was then repeated at the location of another radio source. A histogram of the quantized RSSI of each radio sensor was then analyzed to obtain the occurrence probability of the quantized RSSI. Using the occurrence probability, an optimal mapping was derived under the packet collision probability minimization condition. Therefore, a mapping table was obtained for each sensor corresponding to the locations of the two radio sources. This resulting table was then stored at the GW and each sensor. In the proposed method, one of the two designed mapping tables was selected and used for information gathered by the PLIM, based on the location estimation of radio sources. Two methods were used to estimate the position of the radio source: one used the position information of the sensor with the largest RSSI as the position of the radio source, and the other estimated the position by adding the centers of gravity [28].

The GW estimates the position of the radio source at each frame time, as defined by PLIM. In this experiment, there are two locations where the radio sources were located, each assigned a unique identifier. Subsequently, the location coordinates of the estimated radio sources and the squared distance between the location coordinates of the two radio source placement locations were calculated. The placement location with the smaller distance was set as the most promising radio source location. Subsequently, the location numbers of the estimated radio sources were recorded in a FIFO-type memory. Here, the memory size is 3. The location number with the highest frequency among the recorded location information was estimated as the location where the radio source was emitting, and PLIM was performed using the mapping corresponding to that location. The simulation parameters for Experiment 2 are the same as those used for Experiment 1.

## 6. Experimental Results

Table 2 lists the PDR for Experiment 1. Here, the PDR represents the ratio of the total number of packets transmitted to the number of packets that are successfully demodulated. The terms “optimal mapping”, “random mapping”, and “common mapping” correspond to cases where the mapping is randomly assigned and where all sensors use the same mapping, respectively.

**Figure 9:**
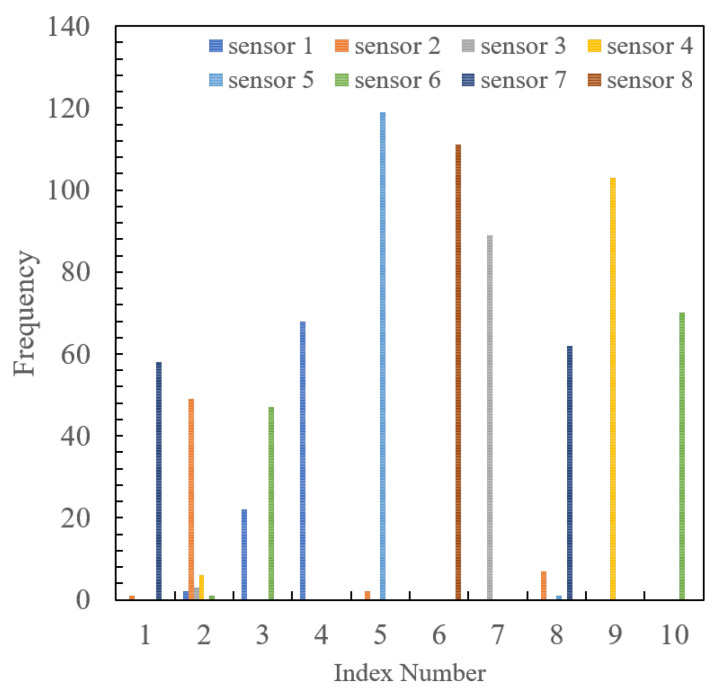
Frequency numbers of indexes selected by each sensor in optimal mapping.

**Figure 10:**
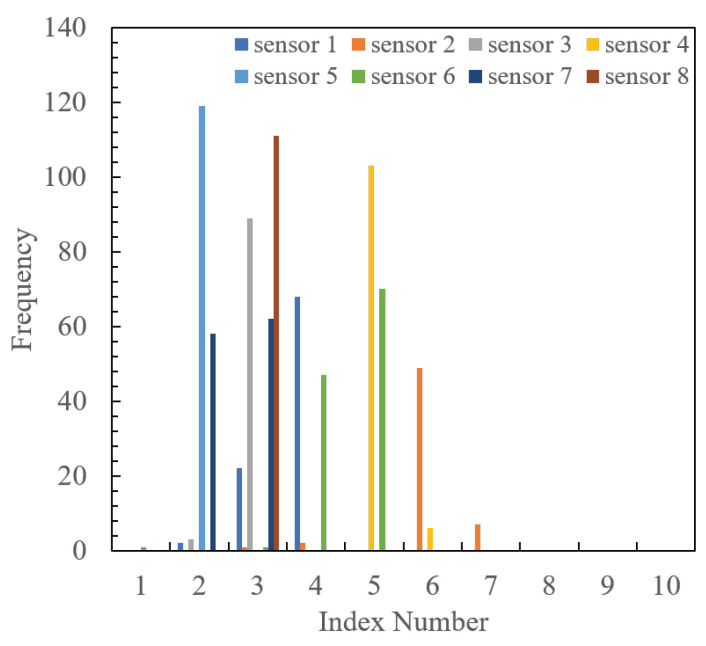
Frequency numbers of indexes selected by each sensor in common mapping.

From the table, it is evident that the optimal mapping achieves a higher PDR than the fixed and random mappings by more than 30%. To clarify the reason behind this, the frequency distribution of index selection made by each sensor is shown in Figure 9 and Figure 10. The former represents the case with the proposed optimal mapping, while the latter depicts the common mapping. From the figures, it is evident that in the fixed mapping, multiple sensors frequently select the same index, resulting in frequent packet collisions. However, the proposed mapping shows that each sensor uses a different index, thereby avoiding packet collisions. Since this experiment was conducted indoors with a single radio source and fixed placement, the RSSI value observed by each sensor remains within a certain range. Therefore, sensors located at the same distance from the source tend to have the same RSSI, leading to frequent packet collisions when selecting the same index in the fixed mapping. Conversely, random mapping is more effective than fixed mapping in avoiding collisions, but it is inferior to the proposed method. Therefore, we confirmed the collision-suppression effect of the proposed method by designing mappings based on minimum collision probability conditions.

Table 3 presents the PDR for Experiment 2. Here, position 1 and 2 indicate the launch positions of the radio source, and the PDR is evaluated for each launch position of each radio source. Position 1 corresponds to Area 1, and Position 2 corresponds to Area 2. The “w/Area Division” column shows the PDR of the PLIM using the mapping optimized by evaluating the probability of RSSI occurrence at position 1 and position 2, respectively. The “ w/o Area Division” shows the PDR of the PLIM using the mapping optimized by evaluating the prior probability of RSSI occurrence for the two emission sources, regardless of their locations. The table shows that the optimal mapping with Area Division achieves the highest PDR for both Positions 1 and 2. Specifically, compared to random mapping, the optimal mapping without Area Division yields an improvement in PDR of approximately 18% to 20%. Conversely, the optimal mapping with Area Division exhibits an improvement from approximately 17% to 19% compared to the optimal mapping without Area Division. Thus, the superior PDR is achieved by harnessing the packet collision-suppression effect of the optimal mapping design and by limiting the launch position of the radio source to specific areas, thereby limiting the probability of RSSI occurrence.

**Figure 11:**
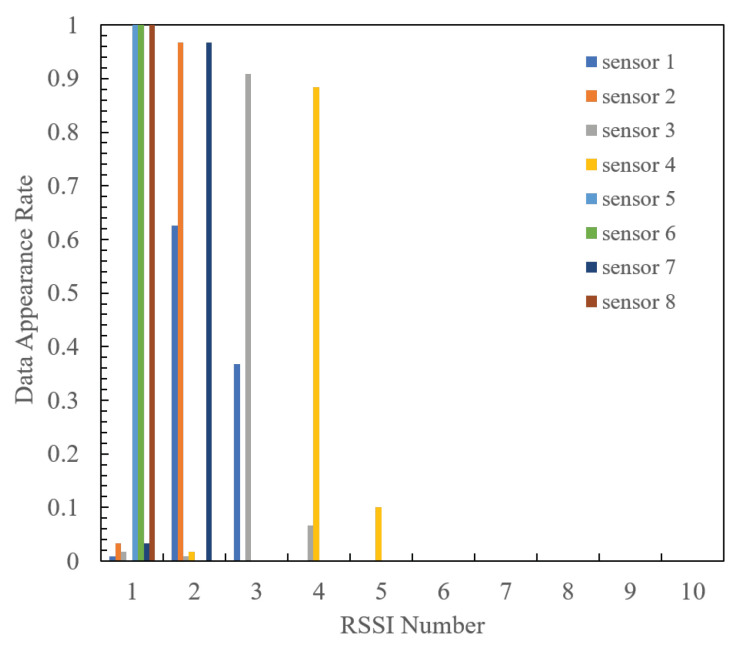
Quantized RSSI generation rates for each sensor in Position 1.

**Figure 12:**
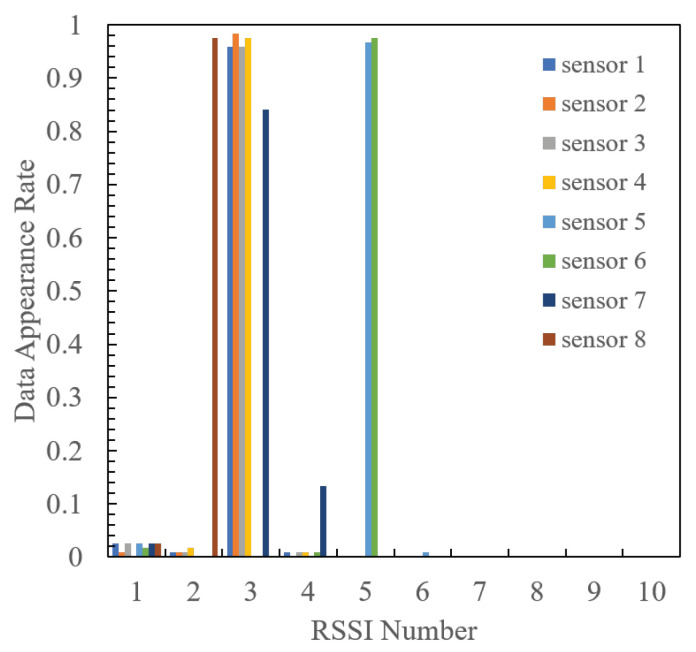
Quantized RSSI generation rates for each sensor in Position 2.

Figure 11 and Figure 12 show the quantized RSSI generation rates for each sensor when the radio source is positioned at 1 and 2, respectively. It is evident that at both radio source positions, each sensor generates a high percentage of specific RSSI values, with a narrow and limited spread of quantized RSSI. Consequently, the optimal design using the proposed method ensures that each sensor transmits high-frequency RSSI at different indices, effectively mitigating packet collisions. Furthermore, the RSSI distribution at position 1 is more widely spread than that at position 2, with a higher proportion of RSSI failing within multiple quantization intervals. This disparity arises because when quantizing RSSI, the RSSI value measured at position 1 corresponds to the boundary of the quantization interval. This may lead to slight fluctuations triggering RSSI quantization switches, thereby obtaining a variety of RSSIs compared to position 2.

Table 4 lists the PDR for each method in Experiment 2. Here, Optimal Mapping refers to the scenario where the optimal mapping is selected based on area estimation derived from the radio source’s location estimation. Nearest Maximal RSSI Sensor indicates the case where the area closest to the sensor, which observed the maximum RSSI, is selected during location estimation. Conversely, Center of Gravity Addition represents the case where location estimation is performed using the method outlined in Ref. [28], and the area nearest to the estimated radio source location is estimated. The table shows that the Nearest Maximal RSSI Sensor achieved a higher gathering success rate than Optimal Mapping without Area Division by 20% and random mapping by 40%. Hence, we have confirmed the improvement of the proposed mapping method, including the location estimation process. However, Optimal Mapping w/Area Division “center-of-gravity addition” degraded by about 6% compared to “Nearest Maximal RSSI Sensor”. This discrepancy can be attributed to the accuracy of the area estimation, where the Nearest Maximal RSSI Sensor exhibited close to 100% accuracy, whereas the Center of Gravity Addition achieved a lower accuracy of 92%. In the nearest maximal RSSI sensor, the position of the sensor measuring the maximal RSSI is considered as the position of the radio source and thus the gap between the actual position of radio source and the estimated one is not smaller than the half of the distance between the two sensors. In the center of gravity addition scheme, as the compensation of positioning the radio sources based on the measured RSSI of each sensor is effective, this gap could be smaller than the maximal RSSI sensor. In the experimental evaluation of this paper, we confirmed the maximal RSSI sensor is better than the center of gravity addition. In this experimental setup, each of the four units was placed in the vicinity of Areas 1 and 2; resulting in a strong relationship between the sensor with the largest RSSI and the Area 2, thereby achieving high estimation accuracy. However, the center of gravity addition tended to use the midpoint between the two separate sensor arrangements as the estimation position, resulting in an indeterminate distinction between areas and degradation of estimation accuracy.

## 7. Conclusions

In this study, we proposed a mapping design for packet-level index modulation (PLIM) that uses pre-observation results of environmental conditions. In a wireless environment where multiple sensors contend for access, packet collisions can result in information loss if the same information is transmitted by PLIM. We introduced a method in which each sensor dynamically switches the mapping, defining the relationship between the index and information assignment, to reduce the probability of packet collision. Our focus was on understanding the change in the statistical trend of sensor information depending on the influencing factor’s locations. Subsequently, we established a method to estimate the location of these factors within a certain range and dynamically switch the mapping according, referred to as an environment-aware adaptive data-gathering method. The effectiveness of our proposed method was verified through computer simulations and actual experiments.

In the proposed method, we use the uniform area division for the initial studies. In the practical situation, the area division depends on the appearance tendency of the event source and thus the suitable area division in accordance with the appearance tendency of the event source is a powerful for additional performance improvement. Therefore, the construction of the suitable area division is important future work.

In the wireless access to the multiple channels, the adjacent channel interference causes the packet drop. This paper tackles the recover of the packet collision under the co-channel wireless access. For clarifying the effect of the proposed scheme, the single channel access is assumed in the experimental evaluation. In practical LPWA, the adjacent channel interference is also serious problem. The recovering of the adjacent channel interference is also important future work.

The adaptation of the proposed data-gathering method to the radio environment has not been evaluated yet. For evaluation, the mobility model of the radio source is required but the suitable model is not determined. The evaluation of the adaptation of the proposed data-gathering method to the radio environment is also important future work.

## Figures and Tables

**Table 1:** Experimental parameters for Experiment 1.

Parameters	Value
Quantization Interval for RSSI	4 [dB]
Number of Indexes	10
Number of RSSI values	10
Minimum RSSI Level	−94 [dBm]
Maximum RSSI Level	−54 [dBm]

**Table 2:** Results of Experiment 1.

Mapping Method	PDR [%]
Optimal Mapping	93.75
Random Mapping	59.58
Common Mapping	51.98

**Table 3:** Experimental results: part 1 of Experiment 2.

Position and Mapping	PDR
Position 1 and Optimal Mapping w/Area Division	69.48 [%]
Position 1 and Optimal Mapping w/o Area Division	50.83 [%]
Position 1 and Random Mapping	30.21 [%]
Position 2 and Optimal Mapping w/Area Division	95.73 [%]
Position 2 and Optimal Mapping w/o Area Division	72.19 [%]
Position 2 and Random Mapping	54.69 [%]

**Table 4:** Experimental results: part 2 of Experiment 2.

Mapping	PDR
Optimal Mapping with Nearest Maximal RSSI Sensor	82.96 [%]
Optimal Mapping with Center of Gravity Addition	76.56 [%]
Optimal Mapping w/o Area Division	61.51 [%]
Random Mapping	42.45 [%]

## Data Availability

The data presented in this study are available on request from the corresponding author.
